# Rooting in the Desert: A Developmental Overview on Desert Plants

**DOI:** 10.3390/genes12050709

**Published:** 2021-05-10

**Authors:** Gwendolyn K. Kirschner, Ting Ting Xiao, Ikram Blilou

**Affiliations:** Plant Cell and Developmental Biology, Biological and Environmental Sciences and Engineering (BESE), King Abdullah University of Science and Technology (KAUST), Thuwal 23955-6900, Saudi Arabia; gwendolyn.kirschner@kaust.edu.sa (G.K.K.); tingting.xiao@kaust.edu.sa (T.T.X.)

**Keywords:** desert plants, cactus, date palm, root system architecture, drought, microbiome, root meristem

## Abstract

Plants, as sessile organisms, have evolved a remarkable developmental plasticity to cope with their changing environment. When growing in hostile desert conditions, plants have to grow and thrive in heat and drought. This review discusses how desert plants have adapted their root system architecture (RSA) to cope with scarce water availability and poor nutrient availability in the desert soil. First, we describe how some species can survive by developing deep tap roots to access the groundwater while others produce shallow roots to exploit the short rain seasons and unpredictable rainfalls. Then, we discuss how desert plants have evolved unique developmental programs like having determinate meristems in the case of cacti while forming a branched and compact root system that allows efficient water uptake during wet periods. The remote germination mechanism in date palms is another example of developmental adaptation to survive in the dry and hot desert surface. Date palms have also designed non-gravitropic secondary roots, termed pneumatophores, to maximize water and nutrient uptake. Next, we highlight the distinct anatomical features developed by desert species in response to drought like narrow vessels, high tissue suberization, and air spaces within the root cortex tissue. Finally, we discuss the beneficial impact of the microbiome in promoting root growth in desert conditions and how these characteristics can be exploited to engineer resilient crops with a greater ability to deal with salinity induced by irrigation and with the increasing drought caused by global warming.

## 1. Introduction

Being sessile, plants are exposed to the continuously changing environment and consequently must adapt their growth behavior to survive the surrounding threats. Desert plants have to cope with additional challenges to adapt to limited water availability and high desert temperatures [1]. Additionally, these plants have to grow and thrive in sandy and infertile soils with low nutrients and high salt content.

Desert plants are categorized into three types based on the shape and morphology of their above-ground organs: (1) annual, with rapid growth and short life cycle (one season); (2) perennial, their survival relies on dormancy during the dry season; and (3) succulents, characterized by a complex and shallow root system that can contain water in the stem [2]. These categories have unique morphological and physiological adaptive traits, particularly the above-ground organs like stem and leaves, which are the most studied organs because they are easily accessible.

A key developmental above-ground trait in desert plants is having smaller and narrower leaves than plants living in temperate habitat, a feature that allows them to limit water loss by transpiration [3]. Many desert plants exhibit high stomata density located on both sides of their leaves and contributing to a higher CO_2_ exchange [3]. Furthermore, their isolateral mesophyll with palisade cells rich in chloroplasts at both leaf surfaces form a large internal surface area, enabling a higher CO_2_ diffusion rate through the cell walls and a higher rate of photosynthesis. In cactus plants, like other desert species, leaves are reduced to spikes. Interestingly, photosynthesis does not occur in leaves but in their thick stems that are flat in some species and round in others. Because of their low surface-to-volume ratio, cacti stems can absorb large amounts of water and, consequently, are considered reservoirs for water storage [3]. These stems are also protected by a thick layer of wax that prevents water loss during transpiration. Being Crassulacean acid metabolism (CAM) plants, cacti have their stomata closed during the daytime and open during the night, which is advantageous as it saves water during the day. Other desert plants like date palms can thrive at high summer temperature by closing their stomata and by having a protective cuticle [4,5].

Desert plants have also adapted their below-ground organs to the dry and infertile soil, their root distribution, topology, morphology and tissue anatomy [6]. Securing a well-developed root system guarantees anchorage in soil and efficient water and nutrient uptake for optimal growth.

In this review, we highlight how desert plants have designed multiple root types to optimize and maximize exploiting soil water reservoirs. We address root adaptations in desert plants at multiscale with a particular focus on two desert plant representatives: *Opuntia ficus indica* (prickly pear) and *Phoenix dactylifera* (date palm) [7,8,9]. Both have developed different strategies to cope with desert conditions and are of high economic importance. Moreover, molecular tools for both species are available: a strategy to stably transform prickly pear was developed, and the date palm genome is publicly accessible [10,11].

## 2. Below-Ground Organs: Rooting in the Desert

Desert soils have heterogeneous water distribution allocated in two reservoirs, one at the surface and the other in deeper soil layers [2]. In most desert species, and with water being a limiting factor, a fast developmental program is put in place for rapid root growth to allow access to water and secure plant survival. One critical process for plants to initiate their life cycle is successful germination, after which they have to tailor their root system according to their surroundings. While most plant species grow out the main embryonic root, the primary root, few species develop seminal embryonic roots. Later in growth, some plants can also produce shoot-borne nodal roots. All root types can develop first-order lateral roots, and eventually, second- and third-order lateral roots [12,13].

The amount of water and its distribution in desert soils is highly unstable and depends on soil depth. In deep soils, the water is recharged by winter and spring precipitations: cooler temperatures reduce evaporation and transpirational water losses. The water then reaches deeper soil layers. In contrast, summer precipitations only replenish the upper soil layers because of the higher evaporation rates caused by elevated temperatures [2].

The root system distribution and architecture in desert plants dictate whether the plant will take up water exclusively from reservoirs at the soil surface, the deep-soil water, or both [2,4,14]. Water source analysis in annual plants and succulent perennials from southern Utah showed that these plants are entirely dependent on summer precipitations accumulated at the soil surface [2,14]. While the herbaceous and woody perennial species use summer and winter-spring precipitation from the deeper soil layers, other perennials rely exclusively on winter precipitations for growth [2]. Similarly, root distribution and water uptake studies of a phreatophyte desert shrub in Northern China showed that this plant utilizes shallow- and deep-water reservoirs for growth. In contrast, the non-phreatophytic desert shrub only uses water from the surface soil areas [15].

**Figure 1:**
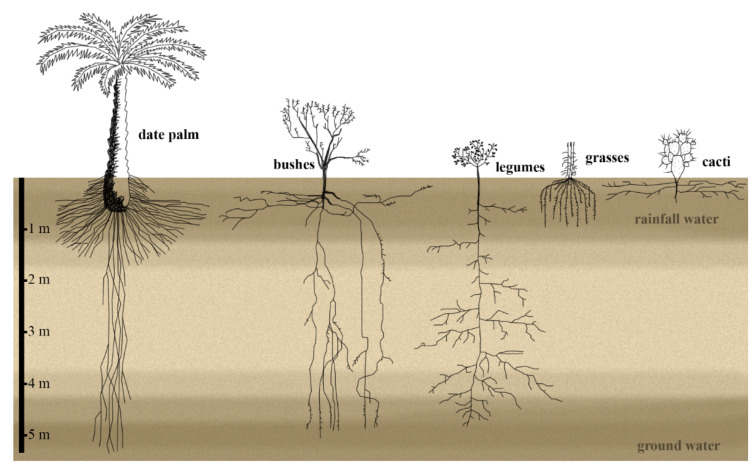
The root system architecture of desert plants exploits the top-soil water reservoir, the deep-soil groundwater, or both. The root system width and shoots size are not according to scale; date palm roots can reach deeper than 5 m [4,7,17,18,19].

Most bushes studied in the Chihuahuan desert exhibit a root system that spreads horizontally from a few to many meters at the shallow soil. Only a few roots penetrate deep soil regions up to 5 m. Among the roots close to the soil surface, some even grow upwards, probably to exploit water resources at the shallow soil in reaction to summer rains [4]. Desert grasses in the Chihuahuan desert have roots with a large lateral spread of 50 to 100 cm in the upper 1.5 m of the soil, most likely to capture the maximum amount of water during rainfalls (Figure 1) [4]. In addition, roots in shallow soils can grow upwards overwriting the normal gravity response [4]. Measuring the hydrotropism response of three Argentinian desert plants revealed that the lateral roots, but not the primary roots, reacted to the water gradient in the soil, indicating that the main roots serve as anchorage in the soil and to exploit the groundwater, while the lateral roots react to rainfall events by hydrotropism and growth towards the water source [16]. In contrast, perennial desert legumes in the Chinese Taklamakan desert develop only primary roots that descend to deeper soil layers, with lateral roots at the deeper soil levels. Only under light irrigation, these plants develop more roots at more shallow soil levels [17]. In summary, the root architecture reflects the soil hydrology by having either a vertically oriented, deep taproot system or horizontally oriented lateral root system (Figure 1).

## 3. The Cactus Root System Is Optimized to Exploit Top-Soil Water

The roots of the cactus Opuntia are mostly distributed in the uppermost 1.5 m of the soil but spread up to 2.5 m from the plant stem horizontally, indicating that these plant species feed on the water reservoir in the upper soil layer [20] (Figure 1). Besides forming primary and lateral roots, the genus Opuntia and the cactus *Stenocereus gummosus* also grow adventitious roots from the shoot to increase anchorage in the soil and water-uptake capacity [21,22]. A large-scale study of the root system architecture of the Cactoideae subfamily of cacti showed that a majority exhibit a primary root with determinate growth and with a length ranging between 1 mm and 60 mm [22]. This determinate growth was observed in lateral and adventitious roots [22]. In different cacti species of the Sonoran Desert, the primary root grows for 2–3 days after germination and differentiates, regardless of water abundance and growth conditions [21]. This growth behavior indicates that root determinacy is not modulated by external cues, but is rather a result of an intrinsic genetic program.

**Figure 2:**
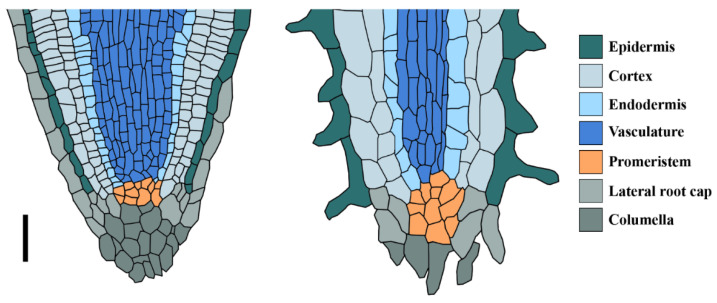
Determinate root growth correlates with cactus meristem differentiation; left: undifferentiated cactus root meristem; right: differentiated meristem with root hairs at the root tip. Root schemes were adapted from real tissue sections described in [33]; scale bar 50 μm.

Root growth relies on the activity of the root apical meristems located at the root tip. The root organ consists of three zones; a meristematic zone containing small, undifferentiated cells that divide actively. These cells leave the meristem shootwards and elongate, forming the elongation zone. Cells above the elongation zone reach their maximum size to form the differentiation zone contributing to longitudinal root growth [23,24,25]. Once mature, root meristems maintain a stable size, by a balance between cell division and differentiation that allows a constant longitudinal root growth [26,27]. This balance is maintained by a combined action of hormone homeostasis involving auxin and cytokinin [26], which is also important for maintaining the stem cell niche through regulating a well-described gene regulatory network [24,28]. The stem cells reside within the root meristem, and the cells derived from them differentiate to form the different tissue types within the root. All stem cells surround the organizer cells, also called the quiescent center (QC) (Figure 2). These cells prevent stem cells from differentiation [29,30,31], and also serve as a pool that replenishes the surrounding stem cells [31,32]. A functional QC is essential for the maintenance of the root meristem and, thereby, root growth. Determinate root growth in cacti correlates with a differentiation of the root meristem, also correlating with a loss of the QC [21,33]. 

This root meristem differentiation process is marked by root hair formation from the epidermal cells close to the root tip and xylem cell differentiation at a certain distance from the root tip [21,34] (Figure 2). This positions mature xylem cells closer to the root tip, allowing rapid water uptake during spontaneous rainfall [34].

Transcriptome analysis of active and differentiated meristems of roots of the cactus *Pachycereus pringlei* identified orthologues of Arabidopsis developmental regulators, such as the GRAS-domain transcription cofactors SHR and SCR, as well as the transcription factor JKD, which regulate ground tissue and QC specification in Arabidopsis [35,36]. This study also revealed an upregulation of genes involved in abscisic acid (ABA) responses at the determinate stage [35]. With the role of ABA in mediating the response to drought, it is plausible that cactus tolerance to drought might be mediated by an increase in ABA levels, while meristem differentiation might result from a change in hormone homeostasis and an alteration of stem cell regulatory networks.

Furthermore, the determinate root growth of the primary root induces the rapid development of lateral roots [21,34]. These lateral roots form a highly interwoven net. The meristem of the first-order lateral roots differentiates quickly, and growth is arrested. The second-order lateral roots form on the first-order ones [21]. Lateral root development is also triggered when the root meristem is lost by physical damage, indicating that the root apical meristem is a source of an inhibitor of lateral root initiation [37,38]. At the same time, the termination of growth in the main roots allocates more nutrients to lateral root growth [38]. It remains to be established which genetic programs induce the rapid meristem differentiation in cactus roots. With this determinate program, cactus roots are becoming an attractive model to study mechanisms of stem cell loss and meristem differentiation.

With auxin and cytokinin determining meristem size and root zonation in other plants, such as Arabidopsis and monocots [26,39], it would be interesting to find out how these two hormones regulate meristem activity in cactus and whether the newly described role of the lateral root cap in determining meristem size could be interpolated to explain the developmental growth in the cactus roots [40,41]. To date, it has only been hypothesized that the phytohormones auxin and cytokinin play a role in maintenance and termination of the cactus root meristem [42]. Analysis of the root apex transcriptome of *Pachycereus pringlei*, showed that orthologues of most genes associated with auxin and cytokinin signaling pathways are present in the transcriptome. Differentially upregulated transcripts in the terminal developmental stage include transcripts for proteins involved in auxin-related processes, such as AUXIN RESPONSE FACTORs and SMALL AUXIN UP RNAs [35]. It would be also informative to determine whether the stem cell loss involves the jasmonic acid regulatory module, similar to Arabidopsis [43].

With its simple meristem organization, implementing mathematical models on cactus root might also help dissecting and understanding their growth dynamics.

Root meristem differentiation might induce lateral roots forming a highly branched root system that covers the soil space horizontally rather than vertically. Although the mechanisms inducing lateral root formation after meristem differentiation in cactus are poorly described, this developmental program is possibly an adaptive response to drought [22]. The determinate root and the formation of the lateral roots in cacti are reminiscent of lateral root arrest upon drought stress in Arabidopsis. In contrast, drought-induced lateral root arrest in Arabidopsis is reversible as the roots can resume growth after rehydration [44].

## 4. Hydraulic Conductivity in Cactus Root Tissue Is Adapted to Drought Conditions

During root growth, meristem cells differentiate shootwards into specific cell types. In young roots, the vasculature is located in the center and is surrounded by the endodermis. The endodermal cells develop the Casparian strip, a hydrophobic fortification of the primary cell wall acting as a diffusion barrier [45]. Next to the endodermis resides the cortex that can consist of one to multilayered files. The cortex is, in turn, surrounded by the exodermis and the epidermis (sometimes called rhizodermis) [45,46]. Adaptation to desert conditions is not only portrayed at the level of the root architecture. It also involves changes at the tissue level to fit the root hydraulic conductivity to these conditions.

In addition to the distribution of their root system, succulents like Opuntia and Agave are also able to develop a so-called rain roots in response to rainfall [20,47,48]. These roots emerge in nodal roots from lateral root buds within a few hours after soil moisture and have high hydraulic conductivities and thereby a higher potential to take up water than established roots. These roots dry out when the water evaporates from surrounding soil.

**Figure 3:**
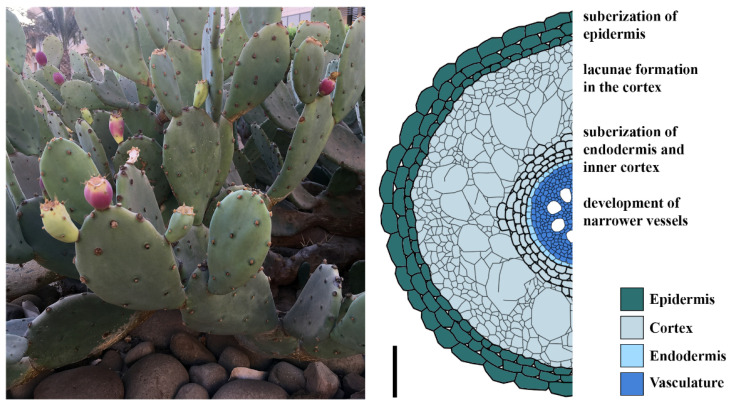
Left: *Opuntia Ficus indica* (prickly pear) in the King Abdullah University of Sciences and Technology (KAUST), Thuwal, Saudi Arabia. Right: schematic representation of a cactus rain root (cross-section); thick cell walls in the epidermis, inner cortex and endodermis indicate suberization; root scheme adapted according to [50,51,55]; scale bar 100 µm.

However, when the soil dries out after rainfall events, water loss through the roots towards the soil has to be prevented, for example by quickly reducing the hydraulic conductivity. One way to lower the hydraulic conductivity in the roots is the formation of lacunae, i.e., air-filled gaps that form from collapsed cells in the cortex, and xylem embolisms. Agave roots develop lacunae, mostly in the rain roots [49]. Similar observations were made in cactus species [50,51] (Figure 3). The correlation between lacuna formation and the decreased hydraulic conductivity was verified by in vivo imaging of grapevine roots under drying conditions. A decrease in hydraulic conductivity correlated with lacuna formation in cortical cells of fine roots under drought conditions. Lacunae formation by the disintegration of cortex cells under drought creates a hydraulic fuse that restricts the tissue damage to internal structures and thereby protects the critical barrier of the epidermis. At the same time, the lacunae act as a resistor in the radial water pathway, limiting water uptake and reflow of water to the drying soil [52]. Moreover, the fine roots are more susceptible to drought-induced xylem embolisms than are coarse roots [52]. These embolisms may prevent the backflow of water from higher plant tissues to the fine roots and its loss in the soil, which has a lower water potential during dry seasons. Furthermore, along with the lacunae formation, Opuntia cactus roots shrink radially in response to drying of the surrounding soil up to 40% in diameter, caused by water loss of the cortical cells, which leads to air gaps between the root surface and soil that help to prevent water loss in the early stage of drought [53,54]. After periods of drought, the young nodal roots of Agave recover their hydraulic conductivity after rewetting, but not the rain roots [49]. These observations explain the short life span of the rain roots that develop in reaction to rainfall and undergo abscission equally fast upon drought [48].

Another way to influence the hydraulic conductivity in roots is the suberization of cell walls. Suberin is a secondary cell wall polymer that forms an apoplastic barrier against water movement and solute flow in plant roots [56]. Roots of Opuntia showed suberization of multiple layers of the epidermis; the number of cell suberized cell layers increased after a prolonged period of drought [50]. Young nodal roots and rain roots of Agave subjected to drought developed suberized cell walls in the exodermis and inner cortex layers adjacent to the endodermis (Figure 3) [49]. Permeability of suberized cells to water is even lower upon drying or exposure to air, preventing the plant from losing water towards the drying soil [50]. Some cactus species are reported to develop narrower vessels in their roots when grown in drought [55]. This might be another mechanism to cope with the negative water potential of dry soil, because smaller vessels are less likely to develop embolisms because of gas spaces.

## 5. Date Palms Adaptation Strategies to Desert Soil

**Figure 4:**
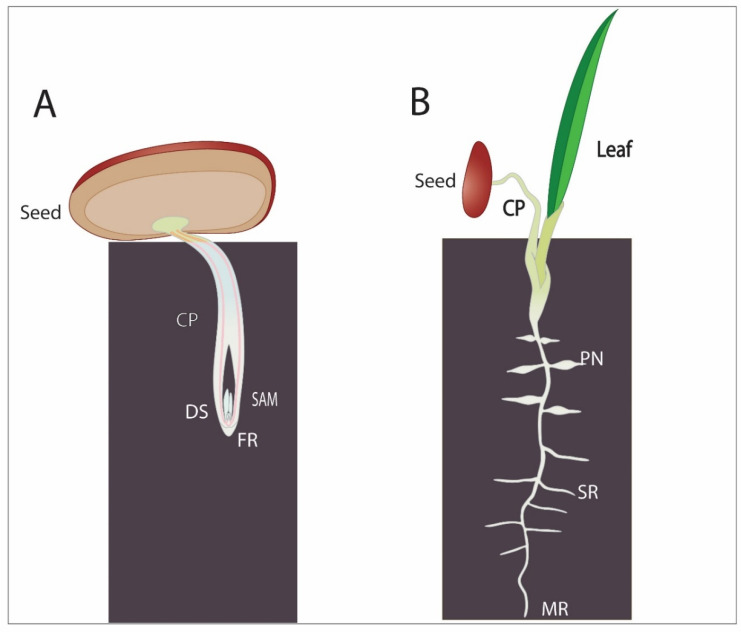
Early developmental processes in date palm. (**A**) Date palm germinating using remote germination. CP: cotyledonary petiole; DS: developing seedling; SAM: shoot apical meristem; FR: future root. (**B**) Date palm seedling: PN: pneumatophores; SR: secondary roots; MR: main roots.

In contrast to cactus’ strategy to grow a horizontally spread root system as fast as possible after germination, date palms have a different approach to protect their seedlings from the harsh desert conditions. They germinate in the so-called “remote-tubular” way (Figure 4A). A root-like structure that consists of the cotyledonary petiole emerges and elongates to transport the embryo and developing seedling away from the surface into the soil. The primary leaf emerges then through an opening from the cotyledonary petiole [9,57]. The tip of the cotyledonary petiole produces the root tip of the future seedling. This structure grows in the soil keeping the developing embryo and the young seedling protected from the heat and dryness at the soil surface while allowing the roots to reach deeper soils [9]. Detailed analysis of the date palm root system revealed a typical root anatomy with the stem cell niche surrounding the QC within the dividing meristem. Transcriptome analysis combined with in situ hybridization assays in date palm root revealed a conservation of the stem cell regulatory network in both roots and shoots. The date palm auxin response gene *INDOLE-3-ACETIC ACID INDUCIBLE2 (PdIAA2)* accumulated in the columella positioning the auxin maxima at this location, while the NAC domain protein SOMBRERO (PdSMB) defined the differentiated columella layers and the lateral root cap [9]. One clear example of network conservation in date palm is the function of the cell fate determinant *SHORT ROOT* (*PdSHR*), that is expressed in the vasculature: ectopic expression of *PdSHR* in Arabidopsis produced additional layers similar to the Arabidopsis and rice orthologues [9]. In addition, *PdSHR* was able to complement the Arabidopsis mutant *shr* when expressed under the Arabidopsis promoter *pSHR* [9]. This emerging evidence highly suggests that stem cell networks are quite conserved between plant species. Establishing genetic tools will bring new insights towards revealing similarities and differences between the date palm and other plant species.

The root system of date palms is adjusted to exploit both the surface-soil water with shallow roots and the groundwater with its vertical roots that can reach deeper than 5 m into the ground [19] (Figure 1). Besides having deep roots, date palms develop pneumatophores, tubular polyp-like roots that grow out from primary or lateral roots close to the soil surface (Figure 4). They can be found above- and below-ground. They contribute to increasing the spatial root distribution to maximize water uptake in response to rainfall events, in addition to condensing water, by growing close to the soil surface [9,58].

**Figure 5:**
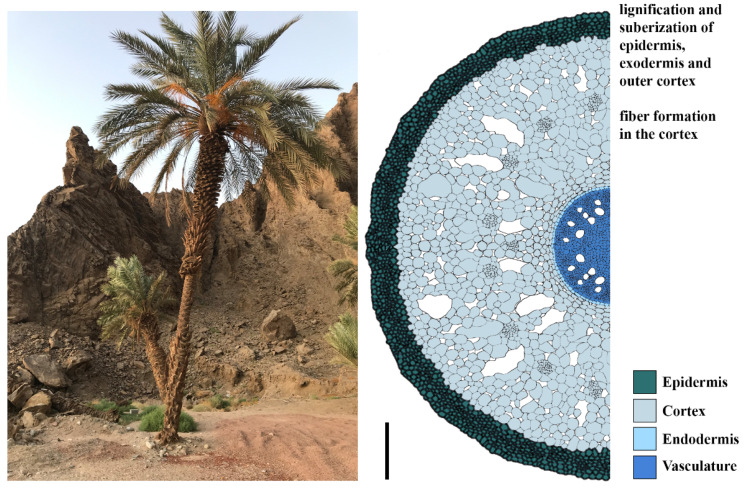
Left:date palm growing at the red sea shore in Tabuk region, Saudi Arabia. Right: schematic representation of a date palm root (cross-section); scale bar 200 µm.

Like in cactus, date palm roots develop suberized barriers, this includes the outer root layers, the epidermis, exodermis, and the outer cortex. The epidermis and exodermis also accumulate lignin to prevent water loss to the soil [9]. In Agave, suberization of the endodermis, the inner cortex cell layers, and the exodermis increased with prolonged drought [49]. Some desert plants also develop suberized fiber cells. In date palms, fiber cells are found within the cortex, while in perennial shrubs, they are located next to phloem cells (Figure 5) [9,59,60]. Close to vessels, fibers most likely serve to protect the water columns from embolism: the presence of fibers around vessels contributes to the cavitation resistance, independently of changing the vessel wall thickness or lumen diameter, and thereby make the roots more stable [61]. Thickened secondary cell walls composed of lignin cellulose and hemicellulose fiber cells provide mechanical support for both roots and shoots to sustain growth [60].

## 6. Desert Microbiome: The Invisible Root Ally

Being the only organ in contact with soil, desert roots, like roots from other habitats, exploit the soil microbiome to increase nutrient uptake, enhance stress tolerance and to protect themselves against pathogens [62]. Recently, there has been an increased focus on microbiomes of desert plants to identify new strains that are selected by the plants to promote their resilience. Interestingly, the composition and nature of these strains are determined among other factors by the soil composition, water content, plant species and their root exudates. Bacterial communities can locate to different compartments that include rhizosphere, rhizosheath and endosphere [63]. A recent report on bacterial communities isolated from different native desert species (*Tribulus terrestris*, *Zygophyllum simplex*, *Panicum turgidum* and *Euphorbia granulate*) identified strains belonging to the Proteobacteria and Enterobacteria with high plant growth promoting potential. These strains promoted root growth and salinity tolerance in *Arabidopsis thaliana* [64,65]. Other studies isolated microbiomes from date palm rhizosphere and showed a predominant presence of bacteria of the genus *Pseudomonas* from the date palm endosphere with a potential to increase plant biomass under water deficiency and enhance phosphate solubilization [66]. Interestingly, the rhizosphere of the desert plant *Alhagi sparsifolia* endosphere also contained *Pseudomonas* strains that increased drought tolerance in wheat [67].

In Arabidopsis, *Pseudomonas spp* promote lateral root formation through modulating auxin signaling and transport [68]. The *Enterobacter* desert sp. induces root hair elongation and improves tolerance to salinity through the ethylene pathway [69]. Thus, root system architecture is impacted by the surrounding microbiome. While the exact mechanism explaining the microbiome contribution to desert plant resilience remains unknown, it is without doubt that root desert plants and their alliance with selected strains provide a foundation for designing unique strategies for sustainable agriculture. Bioengineering stable communities with enough microbial diversity in addition to understanding the molecular mechanism and identifying genes, molecules and pathways governing their action should be the next follow up steps.

## 7. Conclusions: Lessons from Desert Plants

Despite their remarkable adaptability, little attention has been given to studying and understanding the developmental adaptations of desert plant roots. It is noteworthy that there is considerable knowledge that can be learned from these resilient plants. To cope with the dry soil, species like cactus are dedicated to developing a root system in surface soil to tap water from rainfall events. This horizontally oriented root system is formed by the determinate root growth of the main roots, therefore cactus is an interesting model to study root differentiation and stem cell loss. Interestingly, in desert plants like bushes and date palm, roots that grow at the soil surface can grow against the gravity vector towards the soil surface [4,9], probably to react to rainfall events and orient their growth towards the water source. It would be interesting to understand how these roots can balance their hydrotropic and gravitropic growth. In the absence of genetic tools, the new development of 3D imaging technologies and advances in omics at the single cell resolution will open new venues for understanding root adaptations to desert conditions.

For crop plants, developing an ideotype of a root system for different environments can be a promising strategy for improving crop resilience [70]. A hypothetical ideotype for drying soil is the “steep, deep and cheap” root phenotype, in which the primary root grows in a very steep angle deep into the ground to exploit the deep-soil water, and a sub-portion of lateral roots closer at the surface can uptake the top-soil water [71]. While there are many examples of how deeper roots benefit crop plant growth under drought conditions [72], only a limited number of genetic loci controlling root depth are described [73,74]. Genetic and molecular mechanisms for this root growth behavior could be gained from research in desert plants that exhibit the proposed steep, deep, and cheap root architecture, especially in species with available genome information like the date palm [10]. Understanding the mode of action of the desert root microbiome is a promising strategy for bioengineering bacterial communities that can serve as bio-fertilizers and improve plant resilience.

With climate change and increasing desertification threats, understanding rooting in the desert will provide tools that can be used to generate the ideotype roots in future resilient crops.
